# MEXPRESS: visualizing expression, DNA methylation and clinical TCGA data

**DOI:** 10.1186/s12864-015-1847-z

**Published:** 2015-08-26

**Authors:** Alexander Koch, Tim De Meyer, Jana Jeschke, Wim Van Criekinge

**Affiliations:** Department of Mathematical Modeling, Statistics and Bioinformatics, Ghent University, Ghent, Belgium; Laboratory of Cancer Epigenetics, Université Libre de Bruxelles, Brussels, Belgium; Department of Mathematical Modeling, Statistics and Bioinformatics, Faculty of Bioscience Engineering, Ghent University, Coupure Links 653, 9000 Ghent, Belgium

**Keywords:** Oncogenomics, TCGA, Data visualization, Data integration

## Abstract

**Background:**

In recent years, increasing amounts of genomic and clinical cancer data have become publically available through large-scale collaborative projects such as The Cancer Genome Atlas (TCGA). However, as long as these datasets are difficult to access and interpret, they are essentially useless for a major part of the research community and their scientific potential will not be fully realized. To address these issues we developed MEXPRESS, a straightforward and easy-to-use web tool for the integration and visualization of the expression, DNA methylation and clinical TCGA data on a single-gene level (http://mexpress.be).

**Results:**

In comparison to existing tools, MEXPRESS allows researchers to quickly visualize and interpret the different TCGA datasets and their relationships for a single gene, as demonstrated for *GSTP1* in prostate adenocarcinoma. We also used MEXPRESS to reveal the differences in the DNA methylation status of the PAM50 marker gene *MLPH* between the breast cancer subtypes and how these differences were linked to the expression of *MPLH*.

**Conclusions:**

We have created a user-friendly tool for the visualization and interpretation of TCGA data, offering clinical researchers a simple way to evaluate the TCGA data for their genes or candidate biomarkers of interest.

**Electronic supplementary material:**

The online version of this article (doi:10.1186/s12864-015-1847-z) contains supplementary material, which is available to authorized users.

## Background

Over the last few years, large-scale cancer genomics projects have had a significant impact on cancer research. The goal of these projects is to create extensive, publically available and multidimensional oncogenomic datasets using high-throughput technologies. These datasets allow researchers to compare the genomic sequences, epigenetic profiles and transcriptomes of cancer cells to those of normal cells or cells of different cancer (sub)types. The Cancer Genome Atlas (TCGA), a joint effort of the National Cancer Institute and the National Human Genome Research Institute, is an example of such a project (http://cancergenome.nih.gov/).

New findings derived from the statistical and data mining analysis of TCGA data are published regularly and have already proven to be a valuable addition to cancer research [1–4]. Large-scale datasets like TCGA also provide a validation platform for newly identified biomarkers and they are becoming a standard tool for current biomarker research. Another powerful aspect of the TCGA data is the possibility to correlate different types of data. Promoter DNA methylation for example influences gene expression, and aberrant methylation is found in almost every human cancer [5]. The ability to compare these data in a large number of cancer patients is therefore extremely valuable, especially for the identification of DNA methylation biomarkers. Given the growing importance of large-scale datasets for cancer research, intuitive data visualization tools are increasingly crucial to help researchers understand the data, especially when multiple samples and datasets have to be compared.

A number of visualization tools, each focused on one or more specific research questions, are available for TCGA data and offer a wide range of visualization methods [6–9]. There is however no tool available that offers fast and straightforward visualization and interpretation of the expression, methylation and clinical data in TCGA, as well as the relation between these different data types. Such a tool could be of particular use to the large community of clinical researchers without bioinformatics expertise who are looking for a way to explore genes of interest or candidate biomarkers in the TCGA data.

Here we introduce MEXPRESS, an intuitive web tool for the fast and straightforward querying and visualization of the clinical, expression and methylation data in TCGA and the relationship between these datasets on a single-gene level. MEXPRESS was designed after the principles of graphical excellence as described by Edward Tufte [10] to ensure that the complex and multidimensional TCGA data would be presented in a clear, precise and efficient way to the user. It is generally accepted that analysis and visualization tools intended for a broad research audience should be easy to use and should not require computational or bioinformatics expertise [7, 9, 11, 12]. MEXPRESS was therefore developed to have virtually no learning curve, allowing especially clinical researchers to get their results fast without having to invest time in learning yet another tool.

## Implementation

**Fig. 1:**
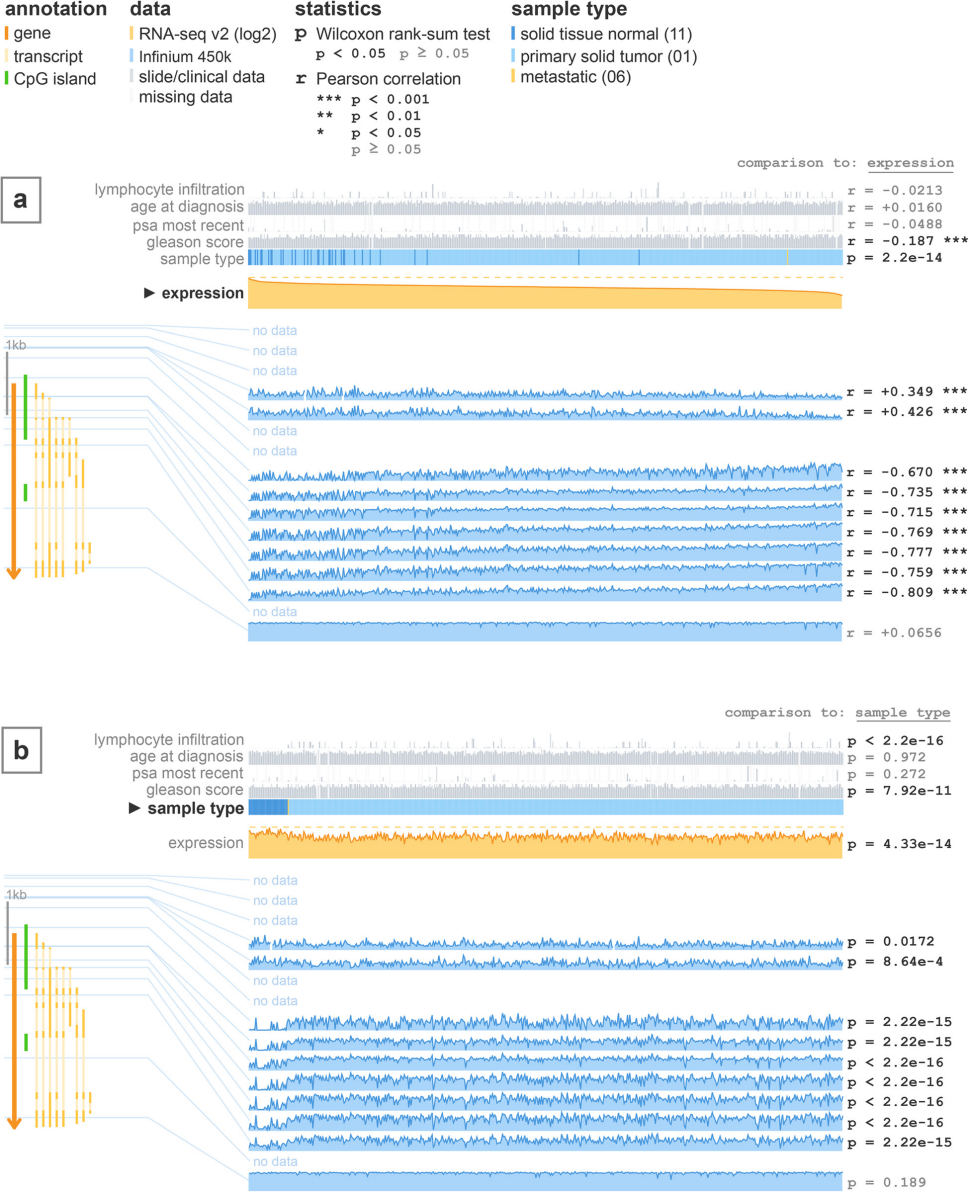
Visualization of the TCGA data for GSTP1 in prostate adenocarcinoma using MEXPRESS. **a** In the default MEXPRESS plot, the samples are ordered by their expression value. This view shows how *GSTP1* expression and promoter methylation are negatively correlated, which is confirmed by the Pearson correlation coefficients on the right. It also indicates that normal samples tend to have higher *GSTP1* expression than tumor samples. **b** When reordered by sample type, the differences in expression and methylation between normal and tumor samples become even more apparent

**Fig. 2:**
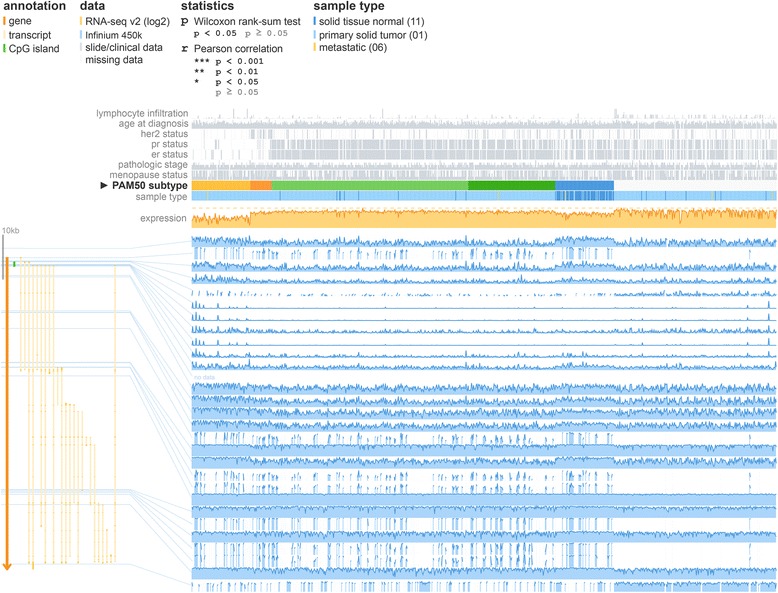
MEXPRESS view of the TCGA data for MLPH in breast invasive carcinoma. The samples are ordered by breast cancer subtype, revealing clear differences in expression and methylation, as well as *HER2*, estrogen and progesterone receptor status, between the different subtypes

Ease of use is a key feature of MEXPRESS. Just three simple steps are needed to create a plot: a user has to enter a gene name, select one of the available cancer types and click the plot button. The resulting figure (Figs. 1 and 2) shows the selected gene together with its transcripts and any CpG islands. Next to the gene, blue line plots illustrate the methylation data for each probe location (Infinium HumanMethylation450 microarray data). A yellow line plot displays the RNA-seq-derived expression data and grey bar plots represent the values of the clinical parameters. The numbers on the far right indicate the significance of the relation (correlation coefficient or P value, depending on the data types compared) between each row of data (clinical, expression or methylation) and the selected “sorter”. By default, expression is the selected “sorter”, which means that the samples are ordered by their expression value. Clicking on one of the clinical parameters will reorder the samples based on the selected variable and the relationships will be recalculated. The resulting images can be downloaded in PNG or SVG file format.

### TCGA data

We downloaded the following TCGA data from the TCGA ftp site: level 3 per-gene RNA-seq v2 expression data (UNC IlluminaHiSeq_RNASeqV2), level 3 DNA methylation data (JHU_USC HumanMethylation450) and clinical data in Biotab format (both clinical patient and tumor sample data). Bash scripts running on the back-end Linux server check the TCGA ftp site monthly for any data updates, which are then automatically uploaded to the database. Whenever TCGA publishes data for new cancer types, these will also be included in MEXPRESS. Before the upload, R scripts (R version 3.0.2) process the data to address missing values, to combine separate files into one where necessary, to reformat the data and to generate SQL scripts for the data upload. The RNA-seq data is log-transformed before being used to draw the plots and only a selection of the most relevant clinical parameters (for which data is available) is shown in the MEXPRESS plots in order to reduce data clutter.

### Other data sources

For the breast invasive carcinoma samples, we downloaded a table with the expression subtype (normal, basal, luminal A, luminal B and Her2) for each sample from the UCSC cancer genome browser [8]. The CpG island data was downloaded from the UCSC genome browser [13] using the table browser with the following settings: clade: Mammal, genome: Human, assembly: Feb. 2009 (GRCh37/hg19), group: Regulation, track: CpG Islands, table: cpgIslandsExt. The exon and transcript annotation was obtained from Ensembl using the BioMart tool (Ensembl Genes 75, *Homo sapiens* genes GRCh37.p13). We designed MEXPRESS in such a way that it will be easy in the future to include new types of data, such as mutation or proteomics data.

### Statistical analyses

We recreated all the statistical functions used in MEXPRESS in Javascript, with the Pearson correlation and the non-parametric Wilcoxon’s rank-sum test being the two main functions. The former is used to compare two types of data that both have more than 2 levels (e.g. expression and methylation data), whereas the latter is used to calculate the difference of a variable between two groups (e.g. the difference in expression between male and female). To correct for multiple comparisons, we included a false discovery rate correction step [14].

### MEXPRESS website

The MEXPRESS site runs on an Apache server and uses PHP to interact with the back-end database. It employs Javascript, the jQuery Javascript library (version 1.11.0), Ajax autocomplete for jQuery (version 1.2.10, https://github.com/devbridge/jQuery-Autocomplete) and the d3.js Javascript library (version 3.0.6, http://d3js.org/) to create the interactive plots and to perform the calculations for the statistical analyses. When a user downloads a figure, the SVG image is converted into a PNG image using Inkscape, an open source vector graphics editor (http://www.inkscape.org/). The backbone of MEXPRESS is a MySQL database that contains the TCGA data needed for the visualizations. PHP scripts handle the database queries, package the results in JSON and send them back to the user. All the MEXPRESS code (back-end, front-end and data processing) can be cloned or downloaded from this GitHub repository: https://github.com/akoch8/mexpress.

## Results and discussion

One of the best-studied examples of epigenetic aberrations in human cancer is the hypermethylation of the *GSTP1* promoter region in prostate cancer, leading to the transcriptional silencing of *GSTP1* [15–17]. Using MEXPRESS, this effect can be observed in the TCGA data. Figure 1a shows the default MEXPRESS plot for *GSTP1* in prostate adenocarcinoma with the samples sorted by their *GSTP1* expression value. It is immediately clear that the normal samples cluster towards higher *GSTP1* expression and that there is a negative correlation between expression and methylation around the promoter region. The *P* value for the comparison of expression between normal and tumor samples (Wilcoxon rank-sum test, *P* = 2.2e-14) and the Pearson correlation coefficients (ranging from −0.670 to −0.769 around the promoter region) confirm the visual interpretation of the data. When the samples are rearranged based on the sample type (normal vs. tumor), this difference in methylation and expression between normal and tumor samples stands out even more (Fig. 1b). It is not possible to create a similar figure that allows a comparable interpretation using one of the existing tools, as they lack the necessary data implementation and/or features, making them less suitable for clinical researchers (Table 1, Additional file 1: Figures S1, S2, S3 and S4).

**Table 1:** A comparison of different tools for the visualization of TCGA data. As illustrated by the Additional file 1: Figures S1, S2, S3 and S4, there are obvious differences between existing tools and MEXPRESS in both the data and the features these tools offer. This table lists the most relevant of these differences, thereby highlighting some of the strengths and weaknesses of each tool. (*CGW* Cancer Genomics Workbench, *IGV* Integrative Genomics Viewer)

	UCSC genome browser	cBioPortal	CGW	IGV	MEXPRESS
All TCGA cancer and data types available	yes	yes	no	no	no
Integration of expression, DNA methylation and clinical data	no	no	no	no	yes
Statistical interpretation of the relationships	no	yes	no	no	yes
Registration and download required	no	no	no	yes	no

*CGW* Cancer Genomics Workbench, *IGV* Integrative Genomics Viewer

Breast cancer is a heterogeneous disease that covers a myriad of subtypes. Each subtype has distinct biological features, leading to differences in clinical outcome and response to treatment. Perou *et al.* [18] were the first to describe breast cancer subtypes based on gene expression patterns and it was found that these subtypes (luminal-like, basal-like, Her2-enriched and normal-like) have significantly different survival times [19]. The classification of breast cancer samples into these subtypes (based on the PAM50 gene signature [20]) is available in MEXPRESS, allowing users to compare expression, methylation and clinical data between the different subtypes. One member of the PAM50 signature is the gene *MLPH*. Using MEXPRESS, it becomes clear that *MLPH* expression is negatively correlated with DNA methylation in the promoter region (a so far unpublished result) and that expression and methylation, as well as *HER2*, estrogen and progesterone receptor status, differ between the breast cancer subtypes (Fig. 2).

Traditional genome browsers, such as the UCSC genome browser [13], present data as horizontally stacked genomic tracks, which is very useful to display different types of location-bound genomic data. This allows users to observe differences within a track or between a limited number of tracks from different samples. MEXPRESS rotates this more traditional “genome browser view” and organizes samples vertically and the different data types horizontally. This simple transformation offers a very different view of the data, resulting in an easier interpretation of the differences between samples than could be achieved through a conventional genome browser, especially when comparing hundreds of samples at the same time. It also allows for the easy comparison of location-bound genomic features, such as DNA methylation, to expression data or clinical information. The combination of this visualization approach with a simple user interface and the strengths listed in Table 1 sets MEXPRESS apart from existing tools when it comes to visualizing and integrating the expression, DNA methylation and clinical TGCA data.

## Conclusion

Along with their expanding size, the value and significance of large-scale oncogenomics datasets will continue to rise in the coming years. This growth creates a need for intuitive and straightforward tools that enable researchers to quickly analyze and visualize the data of interest. The tool presented here offers a unique set of features, including its ease of use and the integrated visualization of different data types over hundreds of samples. It may therefore help to quickly test hypotheses that concern the discovery of DNA methylation or expression-based biomarkers.

## Availability and requirements

**Project name:** MEXPRESS

**Project home page:**http://mexpress.be

**Operating systems:** MEXPRESS can be accessed using any modern desktop browser

**Programming language:** Javascript, PHP, MySQL, R, bash

**Other requirements:** Javascript must be enabled in order to use MEXPRESS. We recommend using a desktop browser; MEXPRESS was not intended to work on mobile devices.

**Any restrictions to use by non-academics:** None

## Additional file

Additional file 1: Figure S1. This file contains all the supplementary figures. **Figure S1.** shows a UCSC Cancer Genome Browser visualization of the *GSTP1* methylation, expression and clinical TCGA data in prostate adenocarcinoma. **Figure S2.** displays a cBioPortal visualization of the correlation between the TCGA expression and methylation data for *GSTP1* in prostate adenocarcinoma. **Figure S3.** depicts a Cancer Genome Workbench view of the TCGA expression data for *GSTP1* in prostate adenocarcinoma. **Figure S4.** shows an Integrative Genomics Viewer visualization of the *GSTP1* expression and methylation TCGA data in glioblastoma multiforme. (DOCX 1513 kb)

## Abbreviation

TCGA: The cancer genome atlas
