# Supplementary material for: MEXPRESS: visualizing expression, DNA methylation and clinical TCGA data
Source: BMC Genomics. 2015 Aug 26;16(1):636. doi: 10.1186/s12864-015-1847-z (PMC4549898; doi:10.1186/s12864-015-1847-z)
Supplement: Additional file 1: Figure S1. — This file contains all the supplementary figures. Figure S1. shows a UCSC Cancer Genome Browser visualization of the GSTP1 methylation, expression and clinical TCGA data in prostate adenocarcinoma. Figure S2. displays a cBioPortal visualization of the correlation between the TCGA expression and methylation data for GSTP1 in prostate adenocarcinoma. Figure S3. depicts a Cancer Genome Workbench view of the TCGA expression data for GSTP1 in prostate adenocarcinoma. Figure S4. shows an Integrative Genomics Viewer visualization of the GSTP1 expression and methylation TCGA data in glioblastoma multiforme. (DOCX 1513 kb) [file 12864_2015_1847_MOESM1_ESM.docx]

**Supplementary Figures for:**

**MEXPRESS: Visualizing expression, DNA methylation and clinical TCGA data**

Alexander Koch^1^, Tim De Meyer^1^, Jana Jeschke^2^, Wim Van Criekinge^1^

^1^Department of Mathematical Modeling, Statistics and Bioinformatics, Ghent University, Ghent, Belgium

^2^Laboratory of Cancer Epigenetics, Université Libre de Bruxelles, Brussels, Belgium

Correspondence should be sent to:

Alexander Koch

Department of Mathematical Modeling, Statistics and Bioinformatics

Ghent University

Coupure Links 653

9000 Gent

Belgium

alexander.koch@ugent.be

**Contents:**

**Supplementary Figure 1** – UCSC Cancer Genome Browser (CGB) visualization of the *GSTP1* methylation, expression and clinical TCGA data in prostate adenocarcinoma as shown in Figure 1.

**Supplementary Figure 2** – cBioPortal visualization of the correlation between the TCGA expression and methylation data for *GSTP1* in prostate adenocarcinoma.

**Supplementary Figure 3** – A Cancer Genome Workbench (CGWB) view of the TCGA expression data for *GSTP1* in prostate adenocarcinoma.

**Supplementary Figure 4** – Integrative Genomics Viewer (IGV) visualization of the *GSTP1* expression and methylation TCGA data in glioblastoma multiforme.

**Supplementary Figure 1** – UCSC Cancer Genome Browser (CGB) visualization of the *GSTP1* methylation, expression and clinical TCGA data in prostate adenocarcinoma as shown in Figure 1.


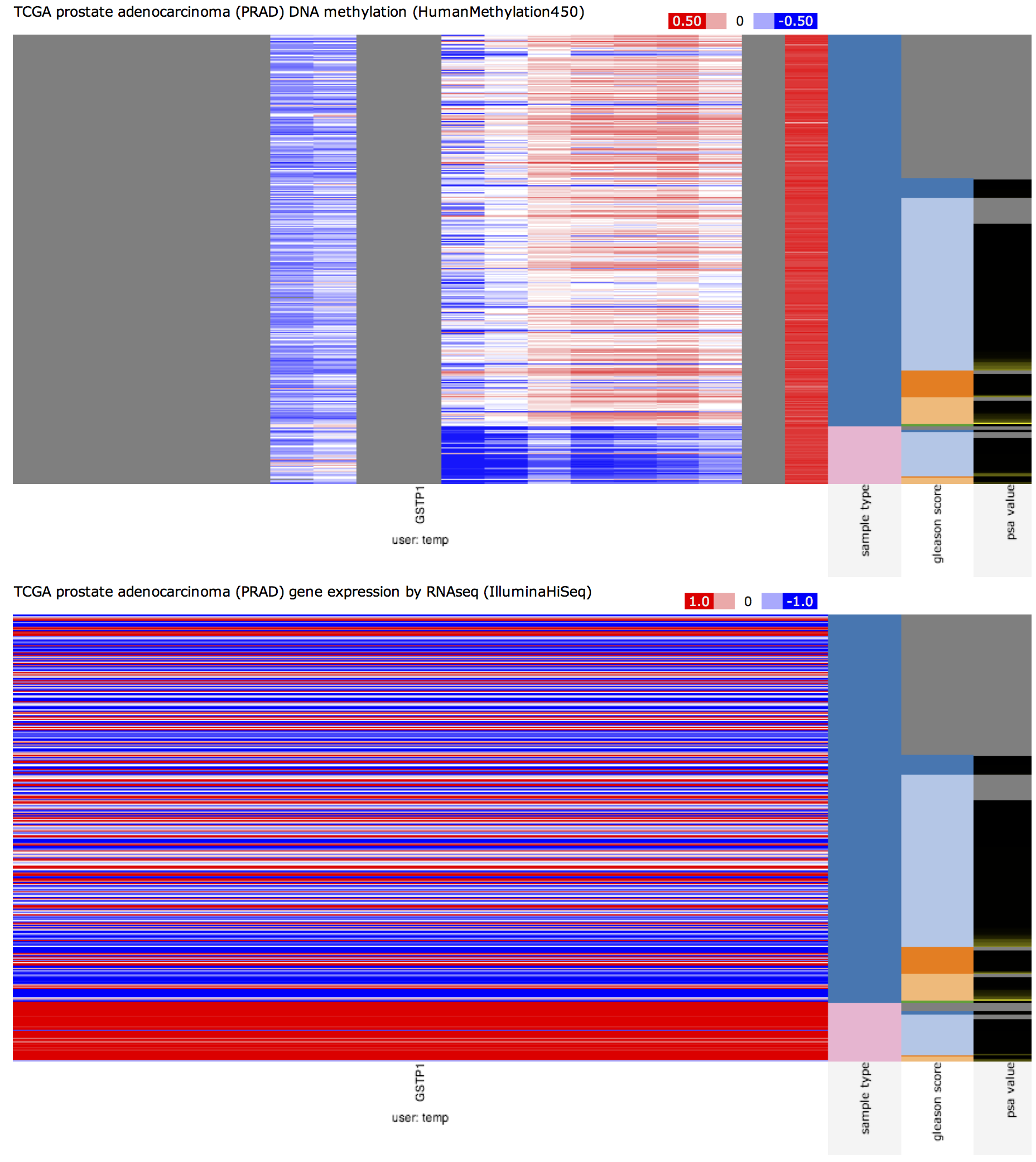


**Supplementary Figure 1** | UCSC Cancer Genome Browser (CGB) visualization of the *GSTP1* methylation, expression and clinical TCGA data in prostate adenocarcinoma as shown in Figure 1. Like MEXPRESS, the CGB allows the samples to be ordered by a clinical parameter, showing differences in methylation and expression between for example the normal and tumor samples. Unlike MEXPRESS however, it is not possible to rank the samples by their expression values or to integrate the expression and methylation data.

**Supplementary Figure 2** – cBioPortal visualization of the correlation between the TCGA expression and methylation data for *GSTP1* in prostate adenocarcinoma.

**Supplementary Figure 2** | cBioPortal visualization of the correlation between the TCGA expression and methylation data for *GSTP1* in prostate adenocarcinoma. Using the cBioPortal tool the correlation between the expression and methylation data for a gene can be visualized, though only for one probe. It is not possible to integrate the expression and methylation data with clinical parameters or to compare the methylation data to the genomic location of the probes as shown in Figure 1.

**Supplementary Figure 3** – A Cancer Genome Workbench (CGWB) view of the TCGA expression data for *GSTP1* in prostate adenocarcinoma.

**Supplementary Figure 3** | A Cancer Genome Workbench (CGWB) view of the TCGA expression data for *GSTP1* in prostate adenocarcinoma. The CGWB is based on the UCSC genome browser and allows a user to plot the expression data for a (limited) number of samples. It offers a more detailed profile of the expression data as compared to the per-gene aggregated expression value shown in MEXPRESS, but cannot integrate the expression profiles with methylation and clinical data.

**Supplementary Figure 4** – Integrative Genomics Viewer (IGV) visualization of the *GSTP1* expression and methylation TCGA data in glioblastoma multiforme.


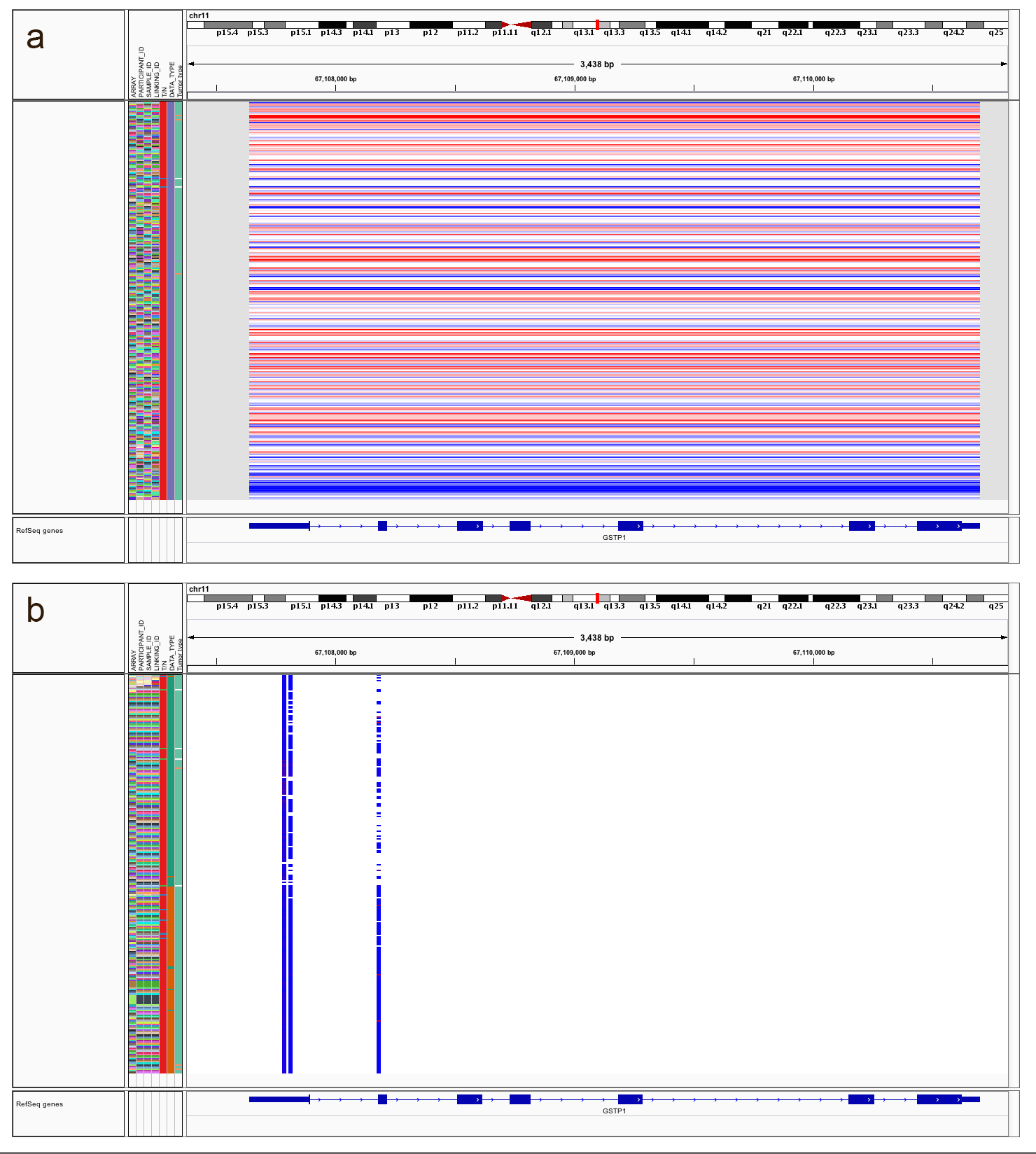


**Supplementary Figure 4** – Integrative Genomics Viewer (IGV) visualization of the *GSTP1* expression and methylation TCGA data in glioblastoma multiforme. The IGV only offers TCGA expression and methylation data for glioblastoma multiforme and ovarian serous cystadenocarcinoma, so no direct comparison could be made to the visualization of the *GSTP1* data in prostate adenocarcinoma as shown in Figure 1. Instead, the *GSTP1* (**a**) expression and (**b**) methylation data is plotted for glioblastoma. The IGV does not offer a direct comparison of the expression and methylation data and does not integrate these datasets with the clinical parameters available in TCGA.
